# Prognostic Value of Sarcopenia in Elderly Patients with Metastatic Non-Small-Cell Lung Cancer Undergoing Radiotherapy

**DOI:** 10.3390/curroncol31110492

**Published:** 2024-10-25

**Authors:** Valerio Nardone, Alfonso Reginelli, Vittorio Patanè, Angelo Sangiovanni, Roberta Grassi, Anna Russo, Pierpaolo Correale, Diego Sandro Giordano, Carmine Zaccaria, Maria Paola Belfiore, Salvatore Cappabianca

**Affiliations:** 1 Department of Precision Medicine, University of Campania Luigi Vanvitelli, 80138 Naples, Italy; alfonso.reginelli@unicampania.it (A.R.); patavittorio@gmail.com (V.P.); angelo.sangiovanni@unicampania.it (A.S.); roberta.grassi@policliniconapoli.it (R.G.); anna.russo@policliniconapoli.it (A.R.); diegosandro.giordano@unicampania.it (D.S.G.); carmine.zaccaria@studenti.unicampania.it (C.Z.); mariapaola.belfiore@unicampania.it (M.P.B.); salvatore.cappabianca@unicampania.it (S.C.); 2 Unit of Medical Oncology, Grand Metropolitan Hospital “Bianchi Melacrino Morelli”, 89128 Reggio Calabria, Italy; pierpaolo.correale@ospedalerc.it

**Keywords:** elderly, morphometry, NSCLC, psoas muscle, sarcopenia

## Abstract

**Background:** Sarcopenia, a syndrome characterized by age-related loss of muscle mass and function, lacks universally accepted diagnostic criteria, particularly for its role as a prognostic factor in elderly patients with non-small-cell lung cancer (NSCLC). This study aimed to evaluate the prognostic significance of sarcopenia, assessed by psoas muscle size on baseline CT scans, in patients over 70 years of age with metastatic NSCLC. **Methods:** We retrospectively analyzed 85 elderly patients undergoing palliative radiation therapy between August 2022 and July 2024. Using morphometric analysis of psoas size, we investigated its correlation with overall survival (OS) and progression-free survival (PFS). **Results:** Our results showed that decreased psoas size was significantly associated with shorter OS and PFS, with median OS of 10 months and PFS of 4 months in sarcopenic patients compared to longer survival times in non-sarcopenic patients. Median survival of non-sarcopenic vs. sarcopenic patients was 21 ± 7 months (muscle area > median) versus 5 ± 2.3 months (muscle area < median). Multivariate analysis confirmed that psoas size, along with ECOG performance status and treatment of primary NSCLC, was a significant predictor of survival. **Discussion:** These findings suggest that psoas muscle size is a valuable prognostic marker for elderly NSCLC patients, potentially guiding treatment decisions and patient management. Further research is needed to validate these results and refine prognostic models for this population.

## 1. Introduction

Sarcopenia, a condition characterized by the progressive loss of muscle mass and strength, is increasingly recognized as a significant prognostic factor in various diseases, particularly among the elderly [1,2,3,4,5]. Officially classified as a distinct disorder in 2016, sarcopenia arises from chronic inflammation, motor neuron atrophy, decreased protein intake often linked to elderly anorexia, and immobility [1,6]. Despite its importance, standardized prevention and treatment guidelines remain underdeveloped [7].

The diagnosis of sarcopenia involves a comprehensive assessment of muscle mass, strength, and physical performance using tools such as grip strength tests, dual-energy X-ray absorptiometry (DEXA), magnetic resonance imaging (MRI), and computed tomography (CT) [8,9,10,11]. CT scans, particularly at the level of the third lumbar vertebra, are commonly employed in preoperative cancer assessments, leading to a focus on CT-determined skeletal muscle index (SMI) in research [12,13,14,15,16].

Sarcopenia significantly increases the risk of adverse outcomes, including higher mortality, functional decline, falls, and hospitalization, in older individuals [17,18,19]. It also serves as a reliable predictor for various cancers, correlating with diminished survival, functional impairment, and heightened chemotherapy toxicity [19,20,21,22,23]. In non-small-cell lung cancer (NSCLC) patients, especially the elderly, sarcopenia has been utilized to predict surgical outcomes and prognosis [24,25,26]. Given the rising incidence of NSCLC among the aging population [27,28,29], this study aims to retrospectively evaluate if a baseline sarcopenic condition can be assessed as a prognostic factor in elderly patients with advanced or metastatic NSCLC [30,31,32,33,34,35]. We hypothesize that patients with sarcopenia will experience shorter survival times.

## 2. Materials and Methods

### 2.1. Patients Population

Between August 2022 and July 2024, 85 patients older than 70 years with metastatic NSCLC underwent radiation therapy (RT) for palliative purposes at our Unit of Radiation Therapy. All patients were consecutively enrolled at the time of the first CT scan confirming the diagnosis of metastatic disease. This includes both patients who were M1 at initial diagnosis (ab initio) and those who were initially M0 but were diagnosed as M1 during follow-up and then. All measurements were taken when the patients were classified as M1. Metastatic disease was confirmed via the first CT scan at diagnosis. Therefore, no patients were M0 at the time of the CT evaluation; all patients had already progressed to M1 status, regardless of whether they were diagnosed with synchronous metastasis (M1 ab initio) or had initially been M0 and later progressed to M1 during follow-up. The staging workup was based solely on CT scans. Brain MRI was reserved as a second-level examination for selected cases where further investigation was required. Only patients without driver mutations who received first-line chemotherapy were selected.

All patients’ clinical and pathological data, collected before RT, were retrospectively recorded, including Karnofsky Performance Status (KPS), use of previous surgery for primary lung cancer, oligometastatic or plurimetastatic disease, technique and localization of RT treatment.

We identified as oligometastatic disease patients with ≤5 metastases in up to three organs [36].

### 2.2. Ethics Approval

All the patients gave their written informed consent to the anonymous use of their examinations for the research’s scope. A study notification was submitted to the local ethical committee as established by national laws. All procedures were undertaken in compliance with the ethical statements of the Helsinki Declaration (2008) of the World Medical Association.

### 2.3. Computed Tomography Imaging

Prior to beginning any kind of therapy, we gathered and examined the initial computed tomography (CT) scan performed at the time of NSCLC diagnosis, whether it be systemic therapy, radiation therapy, or surgery. A 64-detector row CT scanner (Revolution EVO, GE Healthcare, Milwaukee, WI, USA) was used for all CT scans. CT scans of the chest and abdomen were carried out in a tail-cranial orientation on each patient, with the patient laying supine, from the bases of the lungs to a plane that cut through the third of the femur. Following a bolus intravenous infusion of 320 mgI/mL (Ul-travist 320, Schering) and 370 mgI/mL (Iopamiro 370, Bracco, Industria Chimica Milano, Milan, Italy) contrast material at a rate of 4 mL/s, enhanced CT images were acquired. After the bolus, 20–30 mL of saline solution was added, up to a maximum volume of 160 mL, using a power injector (SIAS 757, Milan, Italy) and an 18-gauge needle inserted in the antecubital vein. The scan delay was adjusted to 65–80 s c.a. based on each patient’s functional cardiovascular parameter. A slice thickness of 2.5 mm, beam pitch of 1.375/0.937, reconstruction interval of 0.8 mm, 120–140 kVp, and 250–500 mA were the technical parameters that were employed. The re-construction algorithm was conventional.

### 2.4. Psoas Contouring

**Figure 1:**
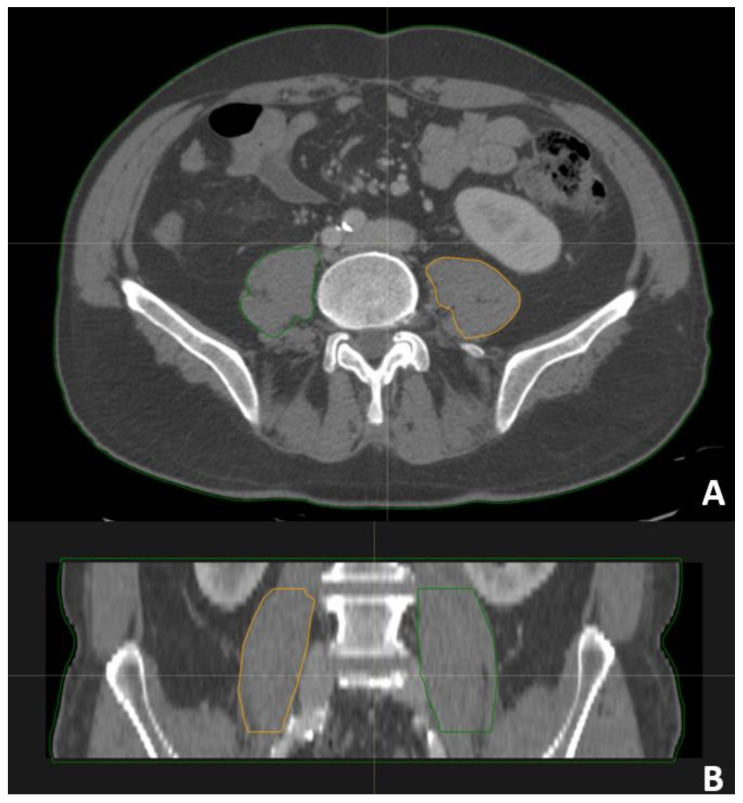
Examples of contouring of psoas muscle. (**A**) Trasversal view; (**B**) coronal view.

Psoas muscle volume, left and right side separately, from the cranial limit of L4 to the caudal limit of L5 vertebral bodies, were calculated in this study (see Figure 1).

To correct for the stature, we utilized a ratio of psoas volume to vertebral body height using the L4 and L5 vertebral body. The rationale is to obtain a mean psoas area (MA) that is considered to be independent on stature [37,38,39].

### 2.5. Radiotherapy

RT was administered as a palliative measure on a case-by-case basis. A Linear Accelerator (6 MV–15 MV photon) photon beam was used to provide the radiation, and treatment planning systems from CMS XiO (Elekta, Sweden) and/or Raystation were utilized to create the RT plan. A diagnostic CT scan was used to determine the target volume. RT dosage was prescribed based on the clinician’s decision in each individual patient. A spiral 16-slice CT scanner with 5 mm slicing, 120 KV, 10 Index Noise, and a range of 100–440 mA was used to run the CT simulation.

### 2.6. Chemotherapy

The cohort of patients underwent different types of chemotherapy regimens, before or after RT, according to the stage of disease, the previous regimens of chemo, and the patients’ clinical status. As previously stated, the patients operated upon at the clinical onset of NSCLC received adjuvant chemotherapy after surgery and are thus considered a distinct subset of this series.

### 2.7. Follow-Up

After the completion of RT, all the patients entered a scheduled follow-up program, and brain CT and MRI scans were repeated at 6 weeks and every 12–16 weeks, or in any case showing clinical signs suggesting progressive disease.

### 2.8. End Points and Statistical Analysis

All the analyses were performed at the first diagnosis of metastatic disease (i.e., at the time of diagnosis for naïve metastatic patients or during the follow-up in patients initially treated for m0 disease).

We assessed the reliability of the volumetric contouring by employing the intra-class correlation coefficient (ICC) method. To do this, we performed double contouring of the muscle area (MA) using two independent operators. Each operator independently contoured the MA on the same set of imaging data, ensuring that the process was blinded to reduce bias. Once the contouring was completed, we compared the resulting variables, including the total muscle area and volume measurements, between the two operators. The ICC was then calculated to evaluate the degree of consistency and agreement between the measurements, providing a quantitative assessment of the inter-operator reliability. This method allowed us to ensure the robustness and reproducibility of the volumetric contouring technique.

The overall survival (OS) was calculated from the date of the patient’s selected CT scan until the patient’s passing or the last follow-up visit. Using the survival analysis (Kaplan–Meier and Cox Rank method), the prognostic parameters associated with the outcome endpoints were determined. The significance of the differences in outcomes was assessed using the log-rank test based on the volumetric parameters that were taken into account and the clinical parameters (KPS, age, sex, oligometastatic status, previous surgery, and RT technique). A *p*-value of <0.05 was deemed statistically significant. The multivariate analysis was performed using the Cox-regression method. Every statistical analysis performed with the SPSS v. 18 Windows software was examined by a biological statistician.

## 3. Results

The main features of our cohort of patients are summarized in Table 1.

### 3.1. Radiotherapy Treatment

All patients underwent palliative treatment for either primary NSCLC or metastatic sites. Specifically, nine patients received palliative treatment for primary NSCLC. The remaining 76 patients were treated for metastatic localizations as follows: 33 patients received palliative treatment for bone metastases, 13 patients underwent whole brain irradiation, 27 patients received stereotactic radiation therapy (for lung, brain, or other sites), and 3 patients were treated for other metastatic localizations.

### 3.2. Clinical Outcome

The median follow-up time was 11 months (mean 24.7 months, SD ± 3.6 months, range 3–36 months). During the follow-up period, 59 patients (70.2%) died. The median overall survival (OS) was 10 months (mean 23.4 months, 95% CI 16–30 months). 

### 3.3. Reliability of Volumetric Parameters and Cut-Offs

The ICC analysis showed that the adopted method of contouring was reliable, achieving an ICC value of 0.94 for the contouring of psoas muscle.

### 3.4. Factors Predicting bRFS and OS

The results of the analysis are summarized in Table 2.

**Figure 2:**
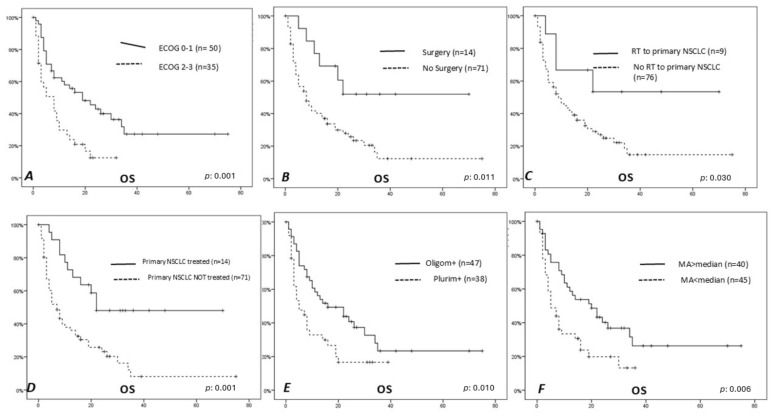
Analysis of survival with Kaplan–Meier method. The parameters that resulted in being significantly correlated with a lower OS were the ECOG ((**A**) *p* < 0.001), previous surgery ((**B**) *p* < 0.001), the number of metastases ((**C**) *p* < 0.001), MA ((**D**) *p*: 0.005), and MA/I ((**E**) *p*: 0.015); patients with mean area (MA) greater than or less than the median ((**F**) *p*: 0.006).

**Figure 3:**
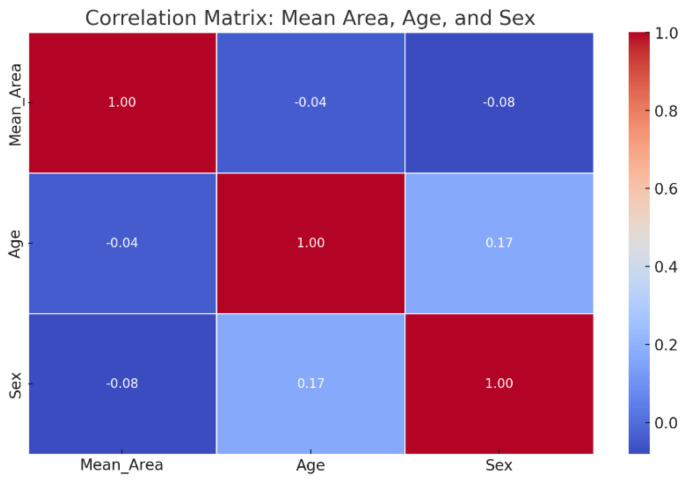
Correlation matrix between mean area (MA), age, and sex. The values within the matrix represent the strength and direction of the correlation between the variables: a value close to 1 indicates a strong positive correlation, a value close to −1 indicates a strong negative correlation, and 0 indicates no correlation. In this chart, we observe the relationship between mean area and demographic variables such as age and sex.

The parameters that resulted in being significantly correlated with a lower OS were the ECOG (*p*: 0.02), MA (*p*: 0.001), previous surgery (*p*: 0.011), number of metastases (*p*: 0.010), RT directed to primary NSCLC (*p*: 0.030), and treatment of primary NSCLC (either with RT or surgery; *p*: 0.001) (see Figure 2 and Figure 3).

In the multivariate analysis, the only parameters that remained significant are as follows: ECOG (*p* < 0.001, OR 2.87, 95%vCI: 1.64–5.03), MA (*p*: 0.001, OR: 0.621, 95%vCI: 0.46–0.83), and treatment of primary NSCLC, either with surgery or RT (*p*: 0.002, OR: 0.33, 95% CI: 0.16–0.66) (see Table 3).

## 4. Discussion

As previously noted, approximately 50% of newly diagnosed cases of non-small-cell lung cancer (NSCLC) occur in patients over the age of 70 [30]. Although mortality rates for NSCLC have been decreasing among younger patients, they continue to rise among the elderly, particularly older women [40,41]. This demographic shift underscores the necessity for treatment decisions in elderly patients to be guided not only by chronological age but also by life expectancy, patient preferences, functional status, comorbidities, and the anticipated benefits and risks of treatment. The loss of skeletal muscle mass and function, a condition known as sarcopenia, is a significant factor that correlates with increased comorbidities and organ function decline in the elderly [42].

This condition not only impacts physical capabilities but also significantly affects prognosis and treatment outcomes. Studies have shown that sarcopenia is a poor prognostic factor in lung cancer, with its presence potentially doubling the risk of mortality [13,23,43,44].

The relationship between sarcopenia and NSCLC is complex, involving immune and metabolic pathways. Sarcopenic patients often experience altered immune responses that can undermine the effectiveness of immunotherapy and exhibit chronic inflammation exacerbating muscle degradation [21,44,45]. Imaging techniques like computed tomography (CT) are commonly used to assess sarcopenia, measuring skeletal muscle area to provide valuable prognostic information [29,46,47].

Sarcopenia, characterized by the loss of skeletal muscle mass and function due to aging, has been increasingly recognized as a significant factor correlating with increased comorbidities and decline in organ function in the elderly [7].

Officially classified as a distinct disease with its own ICD-10 code in 2016, sarcopenia is influenced by chronic inflammation, motor neuron atrophy, decreased protein intake often associated with geriatric anorexia, and immobility [48]. This condition is influenced by chronic inflammation, motor neuron atrophy, decreased protein intake often associated with geriatric anorexia, and immobility [49]. Aging, inflammatory states, neurological disorders, sedentary lifestyles, malnutrition, and iatrogenic factors further exacerbate sarcopenia. Diagnosing sarcopenia involves a comprehensive assessment that includes evaluating muscle strength, muscle mass, and physical performance.

In our study, we examined a cohort of 85 patients over the age of 70 with metastatic NSCLC who underwent palliative radiation therapy (RT) between August 2022 and July 2024. All patients received palliative RT for either primary NSCLC or metastatic sites. The median follow-up period was 11 months, and the median overall survival (OS) was 10 months. The majority of patients (70.2%) passed away during the follow-up period. Our analysis confirmed the reliability of the volumetric contouring method for measuring psoas muscle mass, which was used as a proxy for assessing sarcopenia. Our results identified several factors significantly predicting OS. Higher ECOG performance status scores were associated with reduced OS, indicating that worse functional status correlates with poorer survival outcomes. A lower muscle area (MA) was strongly linked to reduced OS, emphasizing the prognostic value of muscle mass in elderly NSCLC patients. Patients who had undergone surgery for primary NSCLC demonstrated better OS, suggesting that surgical intervention might confer a survival advantage. Additionally, patients with oligometastatic disease had better OS compared to those with plurimetastatic disease. Palliative RT targeting the primary tumor also correlated with improved OS, with the combined treatment of the primary tumor (surgery or RT) significantly enhancing survival.

In multivariate analysis, the significant predictors of OS included ECOG performance status, muscle area, and treatment of the primary NSCLC. Poor performance status independently predicted lower survival. Lower muscle mass remained a significant predictor of poor survival outcomes, and treatment of the primary NSCLC tumor, whether through surgery or RT, independently predicted better survival. These findings highlight the importance of incorporating sarcopenia, as measured by psoas muscle size, into the prognostic evaluation of elderly NSCLC patients. The strong correlation between lower muscle mass and reduced OS underscores the need to include sarcopenia assessment in routine cancer evaluations, especially for the elderly. Moreover, the significant impact of primary NSCLC treatment on survival suggests that aggressive management of the primary tumor may improve outcomes even in metastatic cases.

Sarcopenia, characterized by the loss of muscle mass and function due to aging, is recognized by the European Working Group on Sarcopenia in Older People (EWGOP) as requiring evaluation of both muscle mass and function for accurate diagnosis [10]. While CT and MRI are typically considered gold standards for measurement, there is no consensus on the best method for assessing psoas muscle [6,12]. Despite this, we opted for a volumetric approach to obtain a more reliable measure of muscle mass. Sarcopenia in elderly patients is associated with cancer, overlapping a state of existing muscle loss, and while the correlation with outcomes remains controversial, various mechanisms have been proposed. Skeletal muscle acts as a secretory organ, producing growth hormone and myokines that might suppress oncogenesis and influence immune response [9]. Additionally, inflammatory states, either pre-existing or concurrent with lung cancer, contribute to malnutrition, which in turn leads to catabolic processes and muscle loss [8].

Our study demonstrates that MA, along with clinical parameters, is predictive of OS in elderly NSCLC patients and remains significant in multivariate analysis alongside other prognostic factors such as ECOG status and primary NSCLC treatment. This highlights the added value of muscle measurements in predicting patient outcomes. Other studies have explored similar correlations. For instance, Matsuo et al. found that lower muscle mass was a significant risk factor for non-cancer-related mortality in stage I NSCLC patients undergoing stereotactic body radiotherapy, although no correlation with cancer-related death was observed. Cortellini et al. correlated muscle mass and density with progression-free survival and hematological toxicities in metastatic NSCLC patients undergoing first-line chemotherapy, finding low muscle mass predictive of these outcomes but not overall survival [50]. This discrepancy may be attributed to differences in patient age and the use of predefined cut-off values versus continuous parameters. Nakamura similarly correlated sarcopenia with surgical complications and survival in NSCLC patients undergoing surgery [2].

In this small subset of elderly patients, the treatment of primary NSCLC, whether through surgery or radiation therapy, is strongly associated with improved survival. This result may reflect a selection bias of patients treated with these modalities, although it supports the hypothesis that distant spread is more likely to originate from the primary tumor rather than distant metastases, particularly in oligometastatic cases.

Our study reinforces the multifactorial nature of cancer prognosis in the elderly, where functional status and muscle mass are crucial alongside traditional oncological factors. These findings advocate for comprehensive geriatric assessments as part of standard cancer care to tailor treatment strategies and enhance patient outcomes. While this study provides valuable insights, it has limitations, including its retrospective design and single-institution setting, which may affect the generalizability of the results. Future research should aim to validate these findings in larger, multicenter cohorts and investigate the mechanisms linking sarcopenia with poor cancer prognosis. Additionally, interventional studies are needed to determine whether strategies to preserve or enhance muscle mass can improve survival and quality of life in elderly cancer patients. The treatment of primary NSCLC, whether through surgery or radiation therapy, is strongly associated with improved survival, reflecting the potential impact of aggressive management on metastatic disease. These findings advocate for comprehensive geriatric assessments as part of standard cancer care to tailor treatment strategies and enhance patient outcomes. Future research should validate these findings in larger, multicenter cohorts and investigate mechanisms linking sarcopenia with poor cancer prognosis. Additionally, interventional studies are needed to determine if strategies to preserve or enhance muscle mass can improve survival and quality of life in elderly cancer patients.

## 5. Limitations

We recognize the many limitations of the present study. Firstly, it was a retrospective study based on a single institution’s population. Thus, we could not evaluate the loss of function together with the loss of muscle mass, as recommended by the EWGSOP.

We acknowledge several limitations in our study. First, due to its retrospective design and single-institution setting, we were unable to evaluate the functional loss alongside muscle mass deterioration, as recommended by the EWGSOP. Furthermore, while a dynamic analysis of sarcopenia throughout therapy would provide valuable insights, such an assessment was not feasible within the scope of this study due to the absence of consistent abdominal CT scans before radiation therapy. We agree that sarcopenia progression, evaluated dynamically over time, is critical, and we hope to incorporate such an approach in future prospective studies, which could allow us to track sarcopenia evolution and its potential impact on prognosis. Dynamic evaluations are very pivotal in oncologic patients, as has been previously demonstrated in our studies [51].

Additionally, our cohort consists of elderly patients treated with radiotherapy who might represent a subset with a poorer prognosis. While RT is a common palliative approach in metastatic NSCLC, its prognostic implications remain controversial, as does its relationship with sarcopenia. Another limitation is the lack of access to reference databases of healthy, non-sarcopenic individuals, which would have allowed for comparison and more definitive cut-offs. Without such data, distinguishing whether a small mean area (MA) reflects anatomical variation or sarcopenia progression is challenging. Although we used MA values based on our cohort, this introduces potential bias given that some patients were already sarcopenic at baseline.

Elderly patients, also, should undergo specific geriatric tests and assessments, whereas we collected only simple performance status as ECOG [52]. Finally, NSCLC patients that were treated with RT could represent a subset of patients with a worse prognosis, although the use of RT is very common in metastatic NSCLC, and its correlation with prognosis is still controversial [53,54].

## 6. Conclusions

In conclusion, our findings suggest that MA in NSCLC elderly patients could represent an independent prognosticator of survival and could help to stratify the patient’s prognosis. Further studies are needed to characterize whether morphometrics can successfully predict survival in NSCLC and whether they could be used to tailor the choice of treatment, especially in the particular subset of older people.

## Figures and Tables

**Table 1:** Patient characteristics (*n* = 138). NSCLC: non-single-cell lung cancer, WBI: whole brain irradiation, SRT: stereotactic radiation therapy.

Parameter	Number	Percentage
**Sex** Male Female	63 22	74.1% 25.9%
**Age** 70–79 ≥80	59 26	69.5% 30.6%
**ECOG** 0 1 2 3	35 15 30 5	41.2% 17.6% 35.3% 5.9%
**Previous Surgery for Primary NSCLC** Yes No	14 71	16.5% 83.5%
**Site of RT** Primary NSCLS Bones WBI SRT Other	9 33 13 27 3	10.6% 38.8% 15.3% 31.8% 3.5%
**Load of Metastases** Oligometastatic Disease Plurimetastatic Disease	47 38	55.3% 44.7%

The median age of the patients was 74 years (mean 76, SD ± 5.8, range 70–90). The cohort consisted of 22 females (25.9%) and 63 males (74.1%). ECOG performance status was 0–1 in 50 patients (58.8%) and 2–3 in 35 patients (41.2%). Of the patients, 14 (16.5%) underwent surgery for primary NSCLC, and 14 (16.5%) received primary NSCLC-directed palliative radiation therapy. Additionally, 47 patients (55.3%) were classified as oligometastatic based on the established criteria. Median survival of sarcopenic vs. non-sarcopenic patients was 21 ± 7 months (MA > median) versus 5 ± 2.3 months (MA < median).

**Table 2:** Factors predicting overall survival (OS). Dichotomous variables (sex, load of metastases, previous surgery for primary NSCLC, primary NSCLC treatment) were analyzed with Kaplan–Meier analysis, whereas continuous parameters are analyzed with Cox regression analysis. HR: hazard ratio. MA: mean area (of psoas) obtained as ratio of psoas volume to vertebral body height using the L4 and L5 vertebral body. The rationale is to obtain a mean psoas area (MA) that is considered to be independent on stature, as provided in the previous literature.

Parameter	OS (Median Value)
**Sex** Females Males	*p*-value: 0.55 10 ± 1.6 months 11 ± 3.3 months
**Age**	*p*-value: 0.13 HR: 1.03 95% CI: 0.99–1.07
**ECOG**	*p*-value: 0.02 HR: 2.31 95% CI: 1.36–3.95
**Previous surgery for primary NSCLC** Yes No	*p*-value: 0.011 Median not reached 8 ± 2 months
**Palliative RT to primary NSCLC** Yes No	*p*-value: 0.030 20 ± 6.2 months 8 ± 2.4 months
**Primary NSCLC treated (either surgery or palliative RT)** Yes No	*p*-value: 0.001 22 ± 3.5 months 7 ± 1.5 months
**Load of metastases** Oligometastatic disease Plurimetastatic disease	*p*-value: 0.010 16 ± 6 months 5 ± 1.9 months
**MA**	*p*-value: 0.001 HR: 0.59 95% CI: 0.44–0.78

**Table 3:** Multivariate analysis (Cox regression analysis) of survival. OS: overall survival, MA: mean area, B: beta coefficient, HR: hazard ratio, CI: confidence interval.

Endpoint	Parameter	*p*-Value	B	HR (95% CI)
**OS**	MA	0.001	−0.47	0.62 (0.46–0.83)
	Treatment of primary NSCLC	0.002	−1.10	0.33 (0.16–0.66)
	ECOG	<0.001	1.05	2.87 (1.64–5.03)

## Data Availability

Data are available on request to the corresponding author.

## References

[1] Falcon L.J., Harris-Love M.O. (2017). Sarcopenia and the New ICD-10-CM Code: Screening, Staging, and Diagnosis Considerations. Fed. Pract..

[2] Nakamura R., Inage Y., Tobita R., Yoneyama S., Numata T., Ota K., Yanai H., Endo T., Inadome Y., Sakashita S. (2018). Sarcopenia in Resected NSCLC: Effect on Postoperative Outcomes. J. Thorac. Oncol. Off. Publ. Int. Assoc. Study Lung Cancer.

[3] Recio-Boiles A., Galeas J.N., Goldwasser B., Sanchez K., Man L.M.W., Gentzler R.D., Gildersleeve J., Hollen P.J., Gralla R.J. (2018). Enhancing evaluation of sarcopenia in patients with non-small cell lung cancer (NSCLC) by assessing skeletal muscle index (SMI) at the first lumbar (L1) level on routine chest computed tomography (CT). Support Care Cancer.

[4] Liu D., Wang S., Liu S., Wang Q., Che X., Wu G. (2024). Frontiers in sarcopenia: Advancements in diagnostics, molecular mechanisms, and therapeutic strategies. Mol. Asp. Med..

[5] He J., Luo W., Huang Y., Song L., Mei Y. (2023). Sarcopenia as a prognostic indicator in colorectal cancer: An updated meta-analysis. Front. Oncol..

[6] Tagliafico A.S., Bignotti B., Torri L., Rossi F. (2022). Sarcopenia: How to measure, when and why. Radiol. Med..

[7] Calvez V., Becherucci G., Covello C., Piccirilli G., Mignini I., Esposto G., Laterza L., Ainora M.E., Scaldaferri F., Gasbarrini A. (2024). Navigating the Intersection: Sarcopenia and Sarcopenic Obesity in Inflammatory Bowel Disease. Biomedicines.

[8] Kara M., Kaymak B., Frontera W., Ata A.M., Ricci V., Ekiz T., Chang K.V., Han D.S., Michail X., Quittan M. (2021). Diagnosing sarcopenia: Functional perspectives and a new algorithm from the ISarcoPRM. J. Rehabil. Med..

[9] Voulgaridou G., Tyrovolas S., Detopoulou P., Tsoumana D., Drakaki M., Apostolou T., Chatziprodromidou I.P., Papandreou D., Giaginis C., Papadopoulou S.K. (2024). Diagnostic Criteria and Measurement Techniques of Sarcopenia: A Critical Evaluation of the Up-to-Date Evidence. Nutrients.

[10] Cruz-Jentoft A.J., Bahat G., Bauer J., Boirie Y., Bruyère O., Cederholm T., Cooper C., Landi F., Rolland Y., Sayer A.A. (2019). Sarcopenia: Revised European consensus on definition and diagnosis. Age Ageing.

[11] Cozzolino I., Ronchi A., Messina G., Montella M., Morgillo F., Vicidomini G., Tirino V., Grimaldi A., Marino F.Z., Santini M. (2020). Adequacy of Cytologic Samples by Ultrasound-Guided Percutaneous Transthoracic Fine-Needle Aspiration Cytology of Peripheral Pulmonary Nodules for Morphologic Diagnosis and Molecular Evaluations: Comparison With Computed Tomography-Guided Percutaneous Transthoracic Fine-Needle Aspiration Cytology. Arch. Pathol. Lab. Med..

[12] Yoon J.K., Lee S., Kim K.W., Lee J.E., Hwang J.A., Park T., Lee J. (2021). Reference Values for Skeletal Muscle Mass at the Third Lumbar Vertebral Level Measured by Computed Tomography in a Healthy Korean Population. Endocrinol. Metab..

[13] Feng Y., Cheng X.H., Xu M., Zhao R., Wan Q.Y., Feng W.H., Gan H.T. (2024). CT-determined low skeletal muscle index predicts poor prognosis in patients with colorectal cancer. Cancer Med..

[14] McGovern J., Dolan R.D., Horgan P.G., Laird B.J., McMillan D.C. (2021). Computed tomography-defined low skeletal muscle index and density in cancer patients: Observations from a systematic review. J. Cachexia Sarcopenia Muscle.

[15] Vogele D., Mueller T., Wolf D., Otto S., Manoj S., Goetz M., Ettrich T.J., Beer M. (2024). Applicability of the CT Radiomics of Skeletal Muscle and Machine Learning for the Detection of Sarcopenia and Prognostic Assessment of Disease Progression in Patients with Gastric and Esophageal Tumors. Diagnostics.

[16] Pigneur F., Di Palma M., Raynard B., Guibal A., Cohen F., Daidj N., Aziza R., El Hajjam M., Louis G., Goldwasser F. (2023). Psoas muscle index is not representative of skeletal muscle index for evaluating cancer sarcopenia. J. Cachexia Sarcopenia Muscle.

[17] Papadopoulou S.K. (2020). Sarcopenia: A Contemporary Health Problem among Older Adult Populations. Nutrients.

[18] Malafarina V., Uriz-Otano F., Iniesta R., Gil-Guerrero L. (2012). Sarcopenia in the elderly: Diagnosis, physiopathology and treatment. Maturitas.

[19] Ventura C., Baldassarre S., Cerimele F., Pepi L., Marconi E., Ercolani P., Floridi C., Argalia G., Goteri G., Giovagnoni A. (2022). 2D shear wave elastography in evaluation of prognostic factors in breast cancer. Radiol. Med..

[20] Granata V., Fusco R., Costa M., Picone C., Cozzi D., Moroni C., La Casella G.V., Montanino A., Monti R., Mazzoni F. (2021). Preliminary Report on Computed Tomography Radiomics Features as Biomarkers to Immunotherapy Selection in Lung Adenocarcinoma Patients. Cancers.

[21] Fasano M., Della Corte C.M., Viscardi G., Di Liello R., Paragliola F., Sparano F., Iacovino M.L., Castrichino A., Doria F., Sica A. (2021). Head and neck cancer: The role of anti-EGFR agents in the era of immunotherapy. Ther. Adv. Med. Oncol..

[22] Williams G.R., Dunne R.F., Giri S., Shachar S.S., Caan B.J. (2021). Sarcopenia in the Older Adult With Cancer. J. Clin. Oncol..

[23] Anjanappa M., Corden M., Green A., Roberts D., Hoskin P., McWilliam A., Choudhury A. (2020). Sarcopenia in cancer: Risking more than muscle loss. Tech. Innov. Patient Support Radiat. Oncol..

[24] Mega S., Fiore M., Carpenito M., Novembre M.L., Miele M., Trodella L.E., Grigioni F., Ippolito E., Ramella S. (2022). Early GLS changes detection after chemoradiation in locally advanced non-small cell lung cancer (NSCLC). Radiol. Med..

[25] Granata V., Grassi R., Miele V., Larici A.R., Sverzellati N., Cappabianca S., Brunese L., Maggialetti N., Borghesi A., Fusco R. (2021). Structured Reporting of Lung Cancer Staging: A Consensus Proposal. Diagnostics.

[26] Belfiore M.P., Sansone M., Monti R., Marrone S., Fusco R., Nardone V., Grassi R., Reginelli A. (2022). Robustness of Radiomics in Pre-Surgical Computer Tomography of Non-Small-Cell Lung Cancer. J. Pers. Med..

[27] Jemal A., Ward E.M., Johnson C.J., Cronin K.A., Ma J., Ryerson B., Mariotto A., Lake A.J., Wilson R., Sherman R.L. (2017). Annual Report to the Nation on the Status of Cancer, 1975-2014, Featuring Survival. J. Natl. Cancer Inst..

[28] Fiorelli A., Sagan D., Mackiewicz L., Cagini L., Scarnecchia E., Chiodini P., Caronia F.P., Puma F., Santini M., Ragusa M. (2015). Incidence, Risk Factors, and Analysis of Survival of Unexpected N2 Disease in Stage I Non-Small Cell Lung Cancer. Thorac Cardiovasc. Surg..

[29] Nardone V., Belfiore M.P., De Chiara M., De Marco G., Patanè V., Balestrucci G., Buono M., Salvarezza M., Di Guida G., D’Angiolella D. (2023). CARdioimaging in Lung Cancer PatiEnts Undergoing Radical RadioTherapy: CARE-RT Trial. Diagnostics.

[30] Nardone V., Nanni S., Pastina P., Vinciguerra C., Cerase A., Correale P., Guida C., Giordano A., Tini P., Reginelli A. (2019). Role of perilesional edema and tumor volume in the prognosis of non-small cell lung cancer (NSCLC) undergoing radiosurgery (SRS) for brain metastases. Strahlenther. Onkol..

[31] Insa A., Martín-Martorell P., Di Liello R., Fasano M., Martini G., Napolitano S., Vicidomini G., Cappabianca S., Franco R., Morgillo F. (2022). Which treatment after first line therapy in NSCLC patients without genetic alterations in the era of immunotherapy?. Crit. Rev. Oncol./Hematol..

[32] Ying L., Xu L., Yang J., Zhang Q. (2024). Prognostic significance of CT-determined sarcopenia in older patients with advanced squamous cell lung cancer treated with programmed death-1 inhibitors. Sci. Rep..

[33] Nardone V., Tini P., Pastina P., Botta C., Reginelli A., Carbone S.F., Giannicola R., Calabrese G., Tebala C., Guida C. (2020). Radiomics predicts survival of patients with advanced non-small cell lung cancer undergoing PD-1 blockade using Nivolumab. Oncol. Lett..

[34] Lee D.A., Lee H.J., Kim J., Park K.M. (2024). Association between patients with migraine and sarcopenia: A retrospective study. Medicine.

[35] Xu J., Wan C.S., Ktoris K., Reijnierse E.M., Maier A.B. (2022). Sarcopenia Is Associated with Mortality in Adults: A Systematic Review and Meta-Analysis. Gerontology.

[36] Ashworth A.B., Senan S., Palma D.A., Riquet M., Ahn Y.C., Ricardi U., Congedo M.T., Gomez D.R., Wright G.M., Melloni G. (2014). An individual patient data metaanalysis of outcomes and prognostic factors after treatment of oligometastatic non-small-cell lung cancer. Clin. Lung Cancer.

[37] Zakaria H.M., Basheer A., Boyce-Fappiano D., Elibe E., Schultz L., Lee I., Siddiqui F., Griffith B., Chang V. (2016). Application of morphometric analysis to patients with lung cancer metastasis to the spine: A clinical study. Neurosurg. Focus..

[38] Swanson S., Patterson R.B. (2015). The correlation between the psoas muscle/vertebral body ratio and the severity of peripheral artery disease. Ann. Vasc. Surg..

[39] Sions J.M., Smith A.C., Hicks G.E., Elliott J.M. (2016). Trunk Muscle Size and Composition Assessment in Older Adults with Chronic Low Back Pain: An Intra-Examiner and Inter-Examiner Reliability Study. Pain Med..

[40] Thandra K.C., Barsouk A., Saginala K., Aluru J.S., Barsouk A. (2021). Epidemiology of lung cancer. Contemp. Oncol..

[41] Ottaiano A., Grassi F., Sirica R., Genito E., Ciani G., Patanè V., Monti R., Belfiore M.P., Urraro F., Santorsola M. (2024). Associations between Radiomics and Genomics in Non-Small Cell Lung Cancer Utilizing Computed Tomography and Next-Generation Sequencing: An Exploratory Study. Genes.

[42] Rodrigues F., Domingos C., Monteiro D., Morouço P. (2022). A Review on Aging, Sarcopenia, Falls, and Resistance Training in Community-Dwelling Older Adults. Int. J. Environ. Res. Public Health.

[43] Huang Y., Yuan F., Yang L., Guo H., Jiang Y., Cun H., Mou Z., Chen J., Li C., Zhang Z. (2024). Computed tomography (CT)-based skeletal muscle vertebral-related index to assess low muscle mass in patients with non-small cell lung cancer. Quant Imaging Med. Surg..

[44] Bonomi P.D., Crawford J., Dunne R.F., Roeland E.J., Smoyer K.E., Siddiqui M.K., McRae T.D., Rossulek M.I., Revkin J.H., Tarasenko L.C. (2024). Mortality burden of pre-treatment weight loss in patients with non-small-cell lung cancer: A systematic literature review and meta-analysis. J. Cachexia Sarcopenia Muscle.

[45] Liu Z., Lei T., Guo Y., Zheng C. (2024). The impact of sarcopenia on the efficacy of PD-1 inhibitors in non-small cell lung cancer and potential strategies to overcome resistance. Front. Pharmacol..

[46] Turcott J.G., Miyagui S.M., Gutiérrez Torres S., Cárdenas-Fernández D., Caballé-Perez E., Rios-Garcia E., Cardona A.F., Rolfo C., Arrieta O. (2024). Sarcopenia as a Predictive Factor for Carboplatin Toxicity in Patients with Advanced Non-Small Cell Lung Cancer. Nutr. Cancer.

[47] Nardone V., Correale P., Mutti L., Desideri I., Romeo C., Pastina P., Tagliaferri P., Caraglia M., Reginelli A., Pirtoli L. (2022). Comparing Addition of Radiotherapy in EGFR- and ALK-Positive NSCLC with Brain Metastases: Are We Evaluating the Optimal End Point?. J. Thorac. Oncol. Off. Publ. Int. Assoc. Study Lung Cancer.

[48] Carotti M., Salaffi F., Beci G., Giovagnoni A. (2019). The application of dual-energy computed tomography in the diagnosis of musculoskeletal disorders: A review of current concepts and applications. Radiol. Med..

[49] Salaffi F., Carotti M., Poliseno A.C., Ceccarelli L., Farah S., Di Carlo M., Giovagnoni A. (2023). Quantification of sarcopenia in patients with rheumatoid arthritis by measuring the cross-sectional area of the thigh muscles with magnetic resonance imaging. Radiol. Med..

[50] Cortellini A., Palumbo P., Porzio G., Verna L., Giordano A.V., Masciocchi C., Parisi A., Cannita K., Ficorella C., Bozzetti F. (2018). Single-institution study of correlations between skeletal muscle mass, its density, and clinical outcomes in non-small cell lung cancer patients treated with first-line chemotherapy. Thorac. Cancer.

[51] Nardone V., Reginelli A., Grassi R., Boldrini L., Vacca G., D’Ippolito E., Annunziata S., Farchione A., Belfiore M.P., Desideri I. (2021). Delta radiomics: A systematic review. Radiol. Med..

[52] Akhtar O.S., Huang L.W., Tsang M., Torka P., Loh K.P., Morrison V.A., Cordoba R. (2022). Geriatric assessment in older adults with non-Hodgkin lymphoma: A Young International Society of Geriatric Oncology (YSIOG) review paper. J. Geriatr. Oncol..

[53] Ashrafi A., Akter Z., Modareszadeh P., Modareszadeh P., Berisha E., Alemi P.S., Chacon Castro M.D.C., Deese A.R., Zhang L. (2022). Current Landscape of Therapeutic Resistance in Lung Cancer and Promising Strategies to Overcome Resistance. Cancers.

[54] Gouran-Savadkoohi M., Mesci A., Pond G.R., Swaminath A., Quan K., Wright J., Tsakiridis T. (2023). Contemporary real-world radiotherapy outcomes of unresected locally advanced non-small cell lung cancer. J. Thorac. Dis..

